# A Review on Extrahepatic Manifestations of Chronic Hepatitis C Virus Infection and the Impact of Direct-Acting Antiviral Therapy

**DOI:** 10.3390/v13112249

**Published:** 2021-11-09

**Authors:** Cesare Mazzaro, Luca Quartuccio, Luigi Elio Adinolfi, Dario Roccatello, Gabriele Pozzato, Riccardo Nevola, Maurizio Tonizzo, Stefano Gitto, Pietro Andreone, Valter Gattei

**Affiliations:** 1 Clinical Experimental Onco-Haematology Unit, Centro di Riferimento Oncologico di Aviano (CRO) IRCCS, 33081 Aviano, Italy; vgattei@cro.it; 2 Rheumatology Clinic Department of Medicine (DAME), ASUFC, University of Udine, 34100 Udine, Italy; luca.quartuccio@uniud.it; 3 Unit Internal Medicine, Department of Advanced Medical and Surgery Sciences, Luigi Vanvitelli University of Campania, 80100 Naples, Italy; luigielio.adinolfi@unicampania.it (L.E.A.); riccardo.nevola@unicampania.it (R.N.); 4 Unit of Nefrology and Dialysis, Department of Clinical and Biological Sciences, San Giovanni Bosco Hospital, University of Turin,10092 Turin, Italy; dario.roccatello@unito.it; 5 Department of Clinical and Surgical Sciences, Maggiore Hospital University of Trieste, 34121 Trieste, Italy; gabriele.pozzato@asugi.sanita.fvg.it; 6 Department of Internal Medicine Pordenone General Hospital, 33170 Pordenone, Italy; maurizio.tonizzo@asfo.sanita.fvg.it; 7 Department of Experimental and Clinical Medicine, University of Florence, 50100 Florence, Italy; stefano.gitto@unifi.it; 8 Unit of Internal Medicine and Metabolic Medicine, University Hospital Moidena and Reggio Emilia, 41100 Modena, Italy; pietro.andreone@unimore.it

**Keywords:** cryoglobulinemia, direct-acting antivirals (DAAs), hepatitis C virus, purpura, arthralgia

## Abstract

Extrahepatic manifestations are a feature of chronic hepatitis C virus (HCV) infection. In the course of chronic HCV infection, about 70% of patients have one or more extrahepatic manifestations. The latter are often the first and only clinical sign of infection. Experimental and clinical data support a causal association for many extrahepatic manifestations and HCV infection, which include mixed cryoglobulinemia, non-Hodgkin lymphomas (NHL), cardiovascular disease, insulin resistance, type 2 diabetes, neurological and psychiatric disease and other rheumatic diseases. All these extrahepatic conditions influence the morbidity, quality of life and mortality of HCV-infected patients. Currently, interferon-free therapeutic regimens with direct-acting antiviral agents (DAA) offer the possibility of treatment to almost the entire infected population, irrespective of stage of cirrhosis and associated serious comorbidities, always maintaining a high efficacy and tolerability. Several studies have shown a close association between HCV clearance by DAAs and an improvement or reduction in the risk of extrahepatic manifestations. Patients with HCV after a sustained virologic response (SVR) by DAA treatment have a lower risk than non-responders of developing cryoglobulinemic vasculitis and B-cell non-Hodgkin’s lymphomas. Furthermore, the SVR by DAA also reduces the risk of acute coronary syndrome, cardiovascular disease, insulin resistance and type 2 diabetes, and it improves atherosclerosis. HCV clearance by DAA also improves the quality of life and survival of patients with chronic HCV infection with associated extrahepatic diseases. Thus, DAAs should be initiated as early as possible in HCV patients with extrahepatic manifestations.

## 1. Introduction

It has recently been estimated that 71.1 million people are infected with the hepatitis C virus (HCV) worldwide, with an annual incidence of 1.75 million [1]. Chronic HCV infection causes approximately 400,000 deaths each year, mostly from liver diseases [1]. HCV also causes extrahepatic diseases that contribute to an increased overall mortality. Among the numerous HCV-associated extrahepatic manifestations reported, the most frequent and best characterized are mixed cryoglobulinemia (MC) [2,3], non-Hodgkin’s lymphomas (NHL) [3], cardiovascular diseases [4], nephropathy [5], insulin resistance (IR) and type 2 diabetes mellitus (T2DM) [6], neurological and psychiatric disorders [7], and non-MC rheumatic diseases (Table 1). The extrahepatic manifestations can influence the morbidity, quality of life, and mortality of these patients [8]. Since 2015, HCV therapy has been based on direct-acting oral antiviral drugs (DAA), which are safe and effective in almost 100% of cases. DAA allow for almost all the infected HCV population to be treated, including patients with advanced stages of cirrhosis and serious associated comorbidities, always maintaining a high efficacy and tolerability. Several studies have shown a close link between treatment-induced, sustained virologic response (SVR) for HVC and a lower risk of extrahepatic manifestations, as well as an improvement om existing HCV-related extrahepatic diseases [9].

The review focuses on an update on HCV-related extrahepatic diseases and the effect that elimination of HCV by DAA treatment has in modifying the outcomes and incidence of extrahepatic manifestations.

## 2. HCV and Mixed Cryoglobulinemia (MC)

MC is defined as the presence of cryoglobulins in the serum, which are immune complexes that reversibly precipitate at the cold temperature of 4 °C [10]. Cryoglobulinemia is classified into types I, II and III. In type I, which is rare, immunoglobulins are monoclonal and have been associated with lymphoproliferative disorders. In types II and III, called MC, the immune complex is composed of polyclonal IgG associated with IgM with rheumatoid factor (RF) activity, which is monoclonal in type II and polyclonal in type III. MC types II and III are mainly associated with chronic HCV infections [10]. MC causes systemic vasculitis involving small-sized vessels. Since the discovery of HCV, it has been shown that 90–95% of MCs that were previously defined as “essential” can be attributed to HCV infection [11]. Clinical and experimental evidence suggests a strong association between HCV and MC and support a causal relationship [12]. The clinical features are variables ranging from mild–moderate symptoms (i.e., purpura, asthenia, and arthralgia) to severe symptoms (i.e., leg ulcers peripheral neuropathy, glomerulonephritis, low-grade NHL) and, in rare cases, life-threatening situations [13,14].

Palpable purpura on the leg is the main symptom, noted in 70–80% of patients, and cutaneous ulcers may occur in 3–11% of cases. Arthralgias are reported in 50–70% of cases; joint pains are bilateral and symmetric, non-deforming, and involve mainly knees and hands. Arthritis occurs in less than 10% of cases, and peripheral neuropathy is observed in 40–50% of cases. Neurologic manifestations range from distal sensory to sensory-motor polyneuropathy, which usually manifests as a painful and asymmetric paresthesia that subsequently becomes symmetric. The motor deficit is inconstant and mainly affects the lower limbs; it appears few months after sensory symptoms or simultaneously [15]. Central nervous system vasculitis is rare and can cause stroke, epilepsy, and cognitive impairment [7]. The most frequently observed kidney damage during MC is type I membranoproliferative glomerulonephritis (MPGN), which occurs in about 55% of cases as an acute renal failure associated with varying degrees of proteinuria and microhematuria. Furthermore, it has been reported that in 2–29% of cases, MC can be associated with sicca syndrome, and in 14–36% with Raynaud’s phenomenon. Low-grade indolent NHL, histology prevalent marginal zone lymphoma (MZL) and plasmacytoid lymphoma are observed in 5–15% of patients [13,14]. HCV patients with MC showed a higher morbidity and mortality. Recent studies have shown an overall survival rate at 10 years of 74%, with a significant difference between type III MC (84%) and type II (71%) [14].

Based on the current etiopathogenetic knowledge of MC, a targeted therapy can be conducted based on different approaches: [1] Antiviral therapy; [2] therapy of B lymphocyte depletion; [3] immunosuppressive therapy and [4] anti-inflammatory drugs.

Many studies have evaluated the effect of antiviral therapy, reporting that HCV clearance rate by DAA treatment in patients with MC is similar to those without MC. The main studies, shown in Table 2, have demonstrated that clearance of HCV by DAA therapy in patients with MC is obtained in from 74% to 100% of cases [16,17,18,19,20,21,22,23,24,25,26]. Unlike the virologic response, the clinical and immunological outcomes of antiviral therapy are less efficient. In fact, at the end of treatment, an improvement or disappearance of the signs and symptoms associated with MC vasculitis is observed in about 60% of cases; however, during follow-up, an increase in clinical improvement has been reported in up to 70% of patients (Table 2). Manifestations such as purpura, skin ulcers, arthralgia and myalgia have clinical improvement in 75–100% of cases within 3–6 months of starting DAA treatment. On the other hand, an improvement in peripheral neuropathy associated with MC after antiviral therapy is achieved, on average, in 40% of cases. In patients with MC-related peripheral severe motor sensory neuropathy, no clinical improvement is generally observed after the disappearance of HCV by DAA. However, these patients show clinical and immunological improvements with rituximab (RTX) (Table 2); therefore, it has been suggested that RTX should be considered in patients with severe peripheral motor sensory neuropathy prior to the initiation of antiviral therapy to obtain a timely and effective therapeutic response [15]. In patients with HCV-related MC and nephropathy, treatment with DAAs improves creatinine levels and mild proteinuria in 40% of cases (Table 2). This should be considered of clinical relevance because renal involvement in MC is associated with poor prognosis [27]. In patients with nephropathy with no clinical response to antiviral therapy, treatment with RTX or apheresis has been shown to be effective in improving both immune and renal function [28,29].

After clearance of HCV by the DAA therapy, disappearance of cryoglobulinemia is observed in only 50% of cases, normalization or decrease of serum rheumatoid factor titers in approximately 33%, and normalization of serum C4 levels in about 30% of cases, and their persistence predisposes patients to possible relapses. HCV-related MC and indolent NHL seem to be refractory to DAA treatment, and these patients usually require chemotherapy or anti-CD20 antibody treatment [12,30,31].

## 3. HCV and Non-Hodgkin Lymphoma (NHL)

The first study describing the possible association between HCV and NHL appeared in 1994 [32], and it was followed by several other studies evaluating this topic around the world [33,34]. In these studies, the mean reported the prevalence of HCV infection in patients with NHL was 19.8%, ranging from 8.9 to 37.1%, with the odds ratio (OR) ranging from 2.6 to 4.3 [34].

Epidemiological studies have shown a different worldwide distribution of the prevalent rates of HCV in NHL patients. The highest rates were reported in Italy (20%), while the rates were 6% in other European countries, 14% in Japan, and 11% in the United States [35]. The histological subtypes of NHL most closely associated with HCV infection were MZL, lymphoplasmacytic lymphoma, and diffuse large B-cell lymphoma (DLBCL) [36,37,38]. HCV-associated DLBCL usually develops from indolent lymphoma. Several virus mechanisms determining neoplastic lymphoproliferative diseases include: (a) continuous external stimulation of the lymphocyte receptor by viral antigens and subsequent proliferation, like the Helicobacter-pylori-induced MALT gastric lymphoma; (b) oncogenic effect of the intracellular viral protein associated with HCV replication within B cells; and, (c) the “hit and run” theory, in which intracellular HCV causes gene mutations (e.g., tumor-suppressor gene) with perpetual damage to B-cells [38].

HCV clearance by DAA therapy has been reported to be associated with a possible regression of B-NHL [39]. Table 3 shows the main studies conducted in HCV patients with associated NHL. Importantly, after HCV clearance by DAA therapy, only a small fraction of patients achieve a complete or partial response in both low- and high-grade lymphomas. However, a few months after the conclusion of antiviral treatment, a significant rate of relapse or progression of the hematological disease is observed, and these patients required immuno- or immunochemotherapy [37,40,41,42,43,44,45].

Patients with the MZL histological subtype were reported to exhibit the best therapeutic outcomes with a complete or partial response after HCV clearance by DAA, while the CLL/SLL subtype showed no response to antiviral treatment [37,42]. However, the observation period of patients with complete or partial hematological response after DAA treatment was too short to draw firm conclusions and a longer follow-up is needed to assess overall long-term efficacy. It is important to emphasize that, due to their high efficacy and safety, DAAs can also be used simultaneously with chemotherapy and in any immunological and clinical situation [46]. There is evidence of the possibility of efficiently performing antiviral therapy with DAAs seven in concomitance with high-dose chemotherapy (R-CHOP) without any side effects [46]. As triggering at NHL onset requires long-lasting HCV replication, antiviral therapy should be performed as early as possible in the natural history of HCV infection, that is, before the lymphoproliferative disease becomes independent of viral replication.

## 4. HCV and Kidney

Renal impairment, especially in the more advanced stages, is associated with an increased incidence of HCV infection. On the other hand, HCV infection during kidney transplantation is associated with an increased risk of liver deterioration, allograft failure and patient mortality. HCV-infected dialysis patients have a higher mortality than non-infected patients due to cardiovascular events, which reflect the chronic endothelial damage induced by the virus, sepsis and liver failure [47]. Patients with stage 4–5 chronic kidney disease (glomerular filtration rate: 15–30 mL/min), whatever the stage of renal fibrosis at the renal biopsy, regardless of their possibility of receiving a transplant, and whether or not they are candidates for kidney transplantation, should be given DAAs, since achieving a SVR decreases the risk of both hepatic and extra-hepatic complications. Favorable safety profiles were shown by sofosbuvir- and velpatasvir-based therapy in patients with advanced renal impairment including not only those with glomerular filtration rate < 30 mL/min, but also patients in renal replacement therapy. The virological responses of treatment of HCV with DAAs drugs in patients with late chronic kidney disease are excellent, similar to those in the general population. Retrospective [48] and prospective [49] studies suggest that DAA therapy is associated with amelioration of the glomerular filtration rate in patients achieving HCV clearance. However, a baseline eGFR of less than 60 mL/min/1.73 m^2^ was a negative predictor of improvement in renal function.

### 4.1. HCV and Glomerular Disease

Cryoglobulinemic nephritis (CN) is a unique type of nephropathy, which is undoubtedly pathogenetically related to HCV infection. CN probably occurs in 0.5% of HCV-infected individuals and represents a key factor affecting prognosis. About 90% of subjects with CN were infected with HCV and about 80% had a significant viral load. DRB1*11-positive subjects were found to be more susceptible to renal involvement. However, DRB1*15-positive patients are less prone to developing cryoglobulinemic nephritis [50]. The possible clinical presentations of CN are summarized in Table 4.

Renal biopsy reveals three main patterns. About 80% of patients have a diffuse membranoproliferative glomerulonephritis. Glomerular basement membrane thickening with double contour appearance, endocapillary proliferation with mesangial enlargement and intracapillary accumulation of neutrophils and monoclonal cells, especially monocytes, and luminal obstruction by cryoglobulin (the so-called pseudo-thrombi) are the main histological features. Crescents and necrosis are relatively uncommon. Lesions are more often diffuse. In 10% of patients, the membranoproliferative glomerulonephritis is focal. About 10% of patients have a mesangial proliferative glomerulonephritis.

Immunofluorescence stains are positive for IgM (usually IgM.k). Strong IgM and IgG deposition is observed in the thrombi, while fibrinogens can be detected in the vessel walls [51]. Since the histological pattern can be very different (implying diverse therapeutic approaches), renal biopsy should be performed in all patients with mixed cryoglobulinemia who present with urinary abnormalities or unexplained renal insufficiency. Due to the HCV association, there has been great interest in the effects of antiviral drugs on these patients [51]. Studies conducted in cryoglobulinemia patients have evaluated the impact of HCV clearance by DAAs on renal function, reporting an improvement in serum creatinine, proteinuria, and eGFR, as well as a reduced risk of developing end-stage renal disease [19]. DAAs are expected to substantially modify the incidence of new CN cases. Unfortunately, virus eradication does not completely block the immunological process once it has been established. The B-cells’ clone often persists and the most severe features of cryoglobulinemic vasculitis (CV), especially nephritis, are substantially unaffected [52]. I is still unclear whether these agents have a significant impact on the immune-mediated injury, especially once the immune disorder is established. The MC cohorts treated with DAAs regimens to date [12] did not fully address the subset of patients who really need aggressive treatment. Glucocorticoids and immunosuppressant are still used in severe renal disease and in acute immunological flares [52].

There is limited experience with the use of cyclosporine, azathioprine and methotrexate. Cyclophosphamide has been frequently used, especially in the past, for the treatment of severe CN. Mycophenolate mofetil has replaced cyclophosphamide in several immune-mediated renal diseases including CN. A standard course of DAAs is indicated following immunosuppression, especially with cyclophosphamide, in order to attenuate viral replication [51].

Remarkable results in severe cryoglobulinemic glomerulonephritis have been obtained with RTX. The lymphoma protocol, consisting of 4 weekly infusions of 375 mg/m^2^, was most frequently used. Two more doses at 1-month intervals have been also used (4 plus 2, the so-called “improved protocol”) [52]. RTX was shown to improve or cure glomerulonephritis in 75–90% of cases. Of note, there is no evidence of HCV reactivation following RTX treatment. Recently, a prospective, single-center open study aiming to evaluate the very long-term effects (mean follow-up 72.47 months), of RTX administration in patients with severe CN without antiviral therapy showed that RTX may also be taken into consideration for maintenance in a few subjects with relapsing severe nephritis [52].

### 4.2. Non-Cryoglobulinemic Glomerular Diseases

There is some association between HCV infection and other glomerular and non-glomerular diseases [53]. HCV infection was found to be associated with several glomerulopathies [54], including membranous nephropathy, focal segmental glomerulosclerosis, and fibrillary and immunotactoid glomerulopathies. Thrombotic microangiopathy associated with anti-cardiolipin antibodies is another putative association. In very few cases, viral non-structural protein-3 (NS3) was detected in the glomerular deposits along the capillary walls and in the mesangium [50]. On the other hand, the few previously described cases of non-cryoglobulinemic membranoproliferative glomerulonephritis were probably misleading due to the inadequate techniques used to detect cryoglobulin and the unavailability of electron microscopy examination [51].

## 5. HCV and Atherosclerosis and Cardio-Vascular Disease

In the last two decades, evidence has accumulated on an association between chronic HCV infection, atherosclerosis, and cardiovascular disease. Experimental data provided the pathogenetic basis of this association, and several pro-atherogenic mechanisms, either directly or indirectly caused by the virus, have been proposed. HCV lives and replicates in thrombotic tissue, where it causes a chronic inflammatory reaction that participates in thrombus growth and instability [55]. Endothelial cells express HCV entry receptors and support viral replication. Furthermore, HCV alters endothelial permeability, causes cell apoptosis and induces endothelial dysfunction by promoting the migration and proliferation of smooth muscle cells from the tunica media to the intimal surface [56]. Therefore, the vascular damage induced by the virus underlies the hypothesis of a direct involvement of HCV in the induction of atherosclerosis. In addition, indirect mechanisms of atherosclerosis have been postulated for HCV, such as chronic, low-grade systemic inflammation and activation of T helper cells with the release of pro-atherogenic cytokines and chemokines (e.g., IL-1β, IL-6, TNF-α), which, in turn, induce, at the endothelial level, the expression of VCA M-1. The latter allows for the binding of monocytes and T lymphocytes to the vascular endothelium at the site of atheroma formation, favoring the transmigration of leukocytes towards the vascular endothelial wall and the growth in atherosclerotic plaque (56). Additionally, HCV induces insulin resistance (IR) and T2DM, hyperhomocysteinemia, vitamin D deficiency, endotoxemia, oxidative stress, and fatty liver, all of which are associated with the development of chronic vascular damage [55].

Clinical studies have shown a close association between HCV and both subclinical and clinical atherosclerosis. HCV patients have a significantly higher prevalence of subclinical carotid atherosclerosis (CA) than HCV-negative subjects (56) and a meta-analysis estimated a higher risk of developing CA (OR, 2.27; 95% CI: 1.76–2.94) among HCV-positive patients than non-infected patients [57,58]. Predictors of CA in HCV patients were fatty liver disease, advanced liver fibrosis, and age over 55 years [56]. A direct correlation was also reported between the degree of IR and the stiffness of the arterial wall in patients with HCV [55]. Most studies and a recent meta-analysis support the role of HCV as a risk factor for the development of coronary artery disease (CAD) and myocardial infarction (MI) [59]. An association between HCV and CAD has been documented in a high number of HCV patients undergoing angiography (6.3% vs. 2.0% HCV-negative; OR 4.2, 95% CI: 1.4–13.0) [60] and in a large veteran population (HR 1.25; 95% CI: 1.20–1.20). Patients with detectable HCV-RNA had a significantly higher incidence of coronary heart disease (CHD) events when compared to patients who were only HCV-antibody-positive with no detectable RNA [61]. Another study on HCV-positive patients has shown a higher incidence of cardiovascular events such as MI (RR, 1.13; 95% CI 1.00–1.28), coronary artery bypass graft, percutaneous angioplasty, and congestive heart failure (OR 2.49, 95% CI: 1.04–5.96), compared to HCV-negative subjects [62].

The global impact of HCV-related cardiovascular disease has been estimated to be 1.5 (95% CI: 0.9–2.1) million disability-adjusted life years (DALY) and the majority of those affected are between 55 and 75 years old, underlining an earlier development of cardiovascular diseases in patients with HCV compared to non-infected subjects [62]. HCV positive patients have an increased risk of death from cardiovascular events (OR 1.65; 95% CI: 1.07–2.56) and a 25% higher risk of ischemic stroke, especially in younger patients [63]. The close association between HCV and ischemic stroke was confirmed in a meta-analysis (RR 1.28; 95% CI: 1.18–1.39) [64]. It was also calculated that the cumulative risk of death from cerebrovascular causes in HCV patients is more than double that of uninfected controls (HR 2.18; 95% CI: 1.50–3.16) and correlates with viral load [65]. Patients with HCV also show a 1.43-fold greater risk of peripheral arterial obstructive disease (95% CI: 1.23–1.67) compared to controls [66]. Recent studies evaluating the impact of HCV clearance by DAA on CA and cardiovascular diseases have indirectly confirmed the fundamental role of HCV in the development of these diseases. A prospective study has shown that elimination of HCV causes a significant improvement in or disappearance of CA [67]. Another prospective study has shown that HCV clearance is associated with a significant decrease in the relative risk of developing cardiovascular disease (RR 0.379; *P* = 0.0002), with an annual incident rate reduction of 68%, and it has been estimated that for every 55 treated patients who achieved HCV clearance by DAA therapy, one major cardiovascular event (MI/stroke) was spared [68]. A large retrospective study showed a reduction in the incidence of cardiovascular disease after HCV clearance by DAAs, of 16.3 per 1000 patient-years (95% CI: 14.7–18.0) compared to 30.4 (95% CI: 29.2–31.7) in controls, as well as a lower risk of cardiovascular events (HR 0.87; 95% CI: 0.77–0.98) [69]. In conclusion, experimental and clinical evidence have shown that HCV infection significantly increases the incidence of atherosclerosis, cardiovascular disease, and mortality from cardiovascular events, and that clearance of HCV by DAAs significantly improves atherosclerosis, and reduces cardiovascular events and relative mortality.

## 6. HCV and Type 2 Diabetes (T2DM)

T2DM is among the extrahepatic manifestations most frequently associated with HCV infection and prediabetes is four times more frequent in HCV patients [55]. The pathogenetic mechanism by which HCV induces T2DM is multifaceted, although mainly related to the development of IR, observed in up to 70% of cases, viral genotypes 1 and 4 and levels of HCV RNA [55]. HCV replicates in pancreatic β-cells causing distress which reduces the β-cells reserve. HCV, through its structural and non-structural proteins, primarily the core protein, directly affects insulin signaling pathways such as IRS-1 and IRS-2 [55]. Indirect mechanisms of IR involve HCV-induce: oxidative stress; fatty liver; release of inflammatory cytokines, such as tumor necrosis factor-α; insulin receptor substrate 1; phosphorylation; protein kinase B; upregulation of gluconeogenic genes, such as glucose 6 phosphatase; and phosphoenolpyruvate carboxy kinase 2 [63]. A meta-analysis that analyzed both retrospective and prospective studies showed a higher risk of T2DM among HCV patients than non-infected subjects (OR: 1.68; 95% CI: 1.15–2.45) [70]. Another meta-analysis evaluating recent studies that included 61,843 HCV patients and 202,130 uninfected controls revealed that the prevalence of T2DM was approximately 15% (95% CI: 13–18%) in patients with HCV and 10% (95% CI: 6–15%) in uninfected controls, and that the risk of T2DM was 1.5 times higher in HCV patients (OR 1.58, 95% CI: 1.30–1.86) [8]. The presence of T2DM in HCV patients was associated with an increased risk of decompensated cirrhosis (HR: 3.6; 95% CI: 1.5–8.3; *P* = 0.003), of developing HCC (HR: 3.28; 95% CI: 1.35–7.97) and of congestive heart failure [55]. Recent prospective studies have shown that HCV clearance by DAAs not only leads to up to 90% improvement or regression of IR status, but also improves control of glucose homeostasis in both T2DM and non-T2DM patients and induces a significant reduction in the incidence of T2DM [68,71]. A prospective, multicenter, case-control study, including over 2400 HCV patients followed for a median of 30 months, demonstrated an 81% relative reduction in the risk of developing T2DM in HCV patients treated with DAAs compared to untreated HCV patients, estimating that, for every 15 HCV recovered patients, one T2DM case is saved [68]. Furthermore, a large retrospective study showed a significant reduction in the incidence rate of T2DM from 20.6/1000 person-years in untreated HCV patients to 9.89 in those treated with DAAs (*p* < 0.001) [72]. The data underscore the central role played by HCV in increased risk of developing IR and T2DM and that the elimination of the HCV can reverse IR and prevent the development of T2DM [72].

## 7. HCV and Central Nervous System (CNS)

The pathogenesis of HCV-related neuro-psychiatric manifestations is extremely complex and poorly understood, and includes both local and systemic inflammation, glial activation, metabolic alterations, and vasculitis. A spectroscopy magnetic resonance (MR) study found an incremented choline/creatine ratio, different from what was reported in hepatic encephalopathy, indicating altered brain metabolism [73]. Other spectroscopy MR studies reported reduced N-acetyl-aspartate (NAA)/creatine ratio in brain cortex, indicating altered neural cells functional integrity [74], and an increment in basal ganglia myoinositol, a marker of CNS inflammation and astrocyte activation [75]. The results from positron emission tomography (PET) studies also support an increment in microglial activation in CHC patients with cognitive alteration [76,77]. Glial cell activation may contribute to pro-inflammatory cytokine synthesis and cognitive dysfunction.

Another potential pathophysiologic mechanism consists of brain HCV replication. Autoptic samples of HCV patients, viral RNA was found in the brain at 1000–10,000 lower titles than in hepatocytes [78]. Endothelial cells that constitute the blood–brain barrier (BBB) have shown to be potential sites of CNS entry. HCV replication in endothelial cells can compromise BBB integrity and alter CNS homeostasis and increase local inflammation [79]. An alternative postulated means of HCV entry in the CNS is through peripheral blood mononucleate cells (PBMC). HCV RNA was found in PBMC, which may act as a ‘trojan horse’, transporting HCV into the CNS, crossing the BBB under certain circumstances [80].

Finally, viral RNA was found in astrocytes and microglial cells [81] although these cells do not express viral receptors; therefore, other mechanisms, such as cell–cell transfection, are hypothesized for viral spreading inside the CNS [82].

## 8. HCV and Neuro-Psychiatric Manifestations

### 8.1. Cerebrovascular Accidents and Parkinson Disease

The literature data demonstrate that HCV-positive subjects have a 1.2–2-fold increased risk of cerebrovascular accidents [83,84] that occur at a younger age and have a worse prognosis than those seen in HCV-negative subjects. It has been shown that serum HCV RNA levels correlate with the risk of death by cerebrovascular disease [65]. The mechanisms involved in the development of cerebrovascular disease are immunocomplexes vasculitis and the formation of carotidal atheromatic plaque. The former is thought to be due to mixed cryoglobulinemia, which involves small CNS vessels and may become clinically overt as multi-infarct encephalopathy with confusion, dysphagia, dysarthria, and cognitive alterations [85,86,87]; the latter is favoured by chronic systemic inflammatory state and local viral replication with consequent mitochondrial damage, and ROS production may end up in plaque destabilization and rupture [7]. Epidemiologic studies reported a more accentuated incidence of Parkinson disease in CHC patients: according to the various authors, the risk seems to be about 1.3-fold greater than in the general population [88,89]. Recently, a prospective study has demonstrated that HCV eradication by DAA treatment significantly reduced the risk of incident cerebrovascular stroke [68]. At present, there are no data on the effect of elimination of HCV by DAA therapy on the prevention of new cases of Parkinson’s disease. However, a reduction in the incidence is conceivable based on previous results, which have shown a lower incidence of Parkinson’s disease in patients with chronic HCV infection who received interferon-based antiviral therapy [90].

Interestingly, Gragnani et al. [91] evaluated the impact of DAA therapy on mental disorders according to the presence of CV. In fact, patients with CV display a major risk of mental impairment. Authors prospectively analyzed data from seventy-six patients demonstrating that the achievement of virological eradication led to a decrease in depression and anxiety in both subgroups.

### 8.2. Inflammatory CNS Diseases and Peripheral Neuropathies

HCV infection may sometimes present with acute or chronic phenomena involving CNS, as myelitis or encephalitis. Other described clinical presentations consist of sensitive ataxia, acute partial transverse myelopathy or spastic paraplegia, which may present with recurrent attacks [92]. These phenomena are probably immune-mediated, since no viral RNA was found in the pathologic tissues or cryoglobulins in serum, and treatment with steroid therapy was effective in some cases of demyelinising encepha-lomyelitis [65,93]. Patients with HCV sometimes develop peripheral neuropathies, which were initially attributed to MC, resulting from ischemic phenomena involving the vasa nervorum, but the recent discovery of perineural T-cell infiltrates suggests an immune-mediated pathogenesis. Peripheral neuropathies range from sensitive poly-neuropathy, the most frequent [7], which presents with a symmetrical onset and a chronic course with progressive involvement from small to large caliber fibers [7], to mono-neuropathy, multiplex mono-neuropathy, and, rarely, demyelinating polyneu-ropathies. Autoimmune neuropathies with vascular and perivascular inflammation have been described in non-cryoglobulinemia patients, in which peripheral nerve biopsies reveal HCV RNA fragments within epineural cells near inflammatory infiltrates [93].

A recent study was conducted, which evaluated the impact of HCV clearance on peripheral neuropathy assessed by neurological examination and electroneurography before and after a median follow-up of 10 months after DAA treatment. The authors studied 37 HCV patients with neuropathic pain, 22 with sensory and motor neuropathy, and 32 with depressed reflexes. At the end of the post-DAA treatment follow-up, improvements were observed in both clinical and subclinical peripheral sensorimotor neuropathy [94].

### 8.3. Cognitive Dysfunction, Psychiatric Manifestations and Quality of Life

Mild cognitive dysfunction, particularly involving attention and working memory [95], affects more than 50% of patients with HCV [92], regardless of the degree of liver disease, suggesting a pathogenic role for HCV [93]. Fatigue, understood as physical and mental fatigue, is even more common, and is complained of by 50–80% of subjects with HCV [96]. Another important issue in HCV population is depression, which is five-fold more frequent than in the general population and frequently misdiagnosed. The National Health and Nutrition Examination Survey, involving more than 10,000 people affected by different liver diseases, found an independent association with depression only in HCV-infected patients, deposing in favour of a possible pathogenic role of HCV in the development of depression [97].

Treatment with DAA has been shown to be safe in psychiatric subjects. In a small population, elimination of HCV by DAAs has been shown to improve anxiety and de-pression [98], although another study could not find an impact on anxiety or depression during or after HCV treatment by DAA [99]. Other studies have evidenced the possibility of an improvement in cognitive functions after viral eradication by DAA [100,101]. A recent study evaluated various cognitive areas using psychometric tests comparing patients with treatment-naive HCV or HCV treated with DAA and heathy individuals found that HCV-positive patients performed significantly differently from healthy controls, but no difference was found between untreated HCV and DAA-treated patients. The authors concluded that there is no cognitive improvement after HCV clearance by DAA [102].

A reduced health-related quality of life is reported in HCV patients, which is, in part, related to the frequent occurrence of psychiatric disorders, social stigma and limitations due to the fear of widespread contagion [103]. Clearance of HCV by DAA treatment has a significant positive impact on quality of life [104]. Short-term and long-term follow-up studies showed improvements after HCV clearance by DAA treatment in terms of quality of life, pain, discomfort, anxiety, depression, and quality of sleep, [104,105,106,107].

## 9. HCV and Non-MC Rheumatic Disorders

### 9.1. HCV and Primary Sjögren Syndrome

Sjögren’s syndrome (pSS), defined as presence of xerostomia, xerophthalmia, anti-SSA or anti-SSB antibodies and typical salivary gland histology is rare in patients with HCV infection [108].

An association between chronic HCV infection and pSS has been reported, even if active HCV infection (with positive PCR) is considered an exclusion criterion for pSS in the classification criteria of pSS [109]. The histopathological hallmarks of pSS are sicca syndrome and focal lymphocytic sialadenitis reported in 10–20% and 25% of HCV patients, respectively [110,111,112,113]. The possible link between pSS and HCV is supported by the presence of the virus in human salivary glands, where it is able to replicate. HCV-related sicca syndrome (SS) presents usually with higher-frequency cryoglobulins, RF, hypocomplementemia and a lower frequency of anti-Ro/SSA-La/SSB. HCV-related SS usually have only mild damage to the glandular tissue [114,115]. HCV-infected patients with sicca syndrome usually report low titers of antinuclear antibodies (ANA) and RF positivity; otherwise, they would rarely have anti-SSA and anti-SSB autoantibodies 2. HCV-related “sicca-like” symptoms tend to be milder compared to pSS [114,115]. Nevertheless, HCV-associated SS is difficult to differentiate from pSS in most cases [113,114].

Sicca/pSS syndrome should be sought in patients with HCV, particularly in those with high titers of RF, or cryoglobulinemia, hypocomplementemia, and anti-Ro/SSA, ad ti-La/SSB [111]. At present, no conclusive data are available on the possible positive effect of HCV clearance by DAA treatment in patients with pSS.

### 9.2. HCV and Arthritis

HCV infection has been reported to be frequently associated with myalgia, fibromyalgia, poly/dermato-myositis (PM/DM), inclusion body myositis (IBM), polyarteritis nodosa (PAN), Behçet’s syndrome, systemic lupus erythematosus (SLE) and antiphospholipid syndrome (APS) [116].

**HCV and PAN.** Following CV, PAN is a frequent systemic vasculitis associated with chronic HCV infection. Patients with HCV-PAN, compared to those with HCV-associated MC, showed a higher frequency of fever, weight loss, severe hypertension, gastrointestinal involvement, severe multifocal acute sensorimotor mononeuropathy, renal and hepatic microaneurysms, and increased levels of protein C-reactive [117].

**HCV and PM/DM.** The possible association between HCV and PM/DM was reported in individual observations. However, HCV has been associated with inflammatory myopathies, mainly PM [118].

**HCV and Myalgia.** About 15% of HCV patients complain of myalgia and the finding of HCV in muscle fibers leads to a pathogenetic involvement of the virus, while conflicting results have been reported for the association between fibromyalgia and HCV [119,120].

**HCV and Osteosclerosis.** This is a rare condition defined as an acquired, painful skeletal disorder characterized by a marked increase in bone mass. It has been described in 22 cases with HCV infection. It has been suggested that HCV, alone or in combination with other unknown agents, can infect and alter bone cells or their precursors in susceptible individuals [121,122].

**HCV and autoantibodies.** The prevalence of circulating autoantibodies is high in HCV-infected patients. The most common is RF, which is present in 70% of patients, from 20% to 40% of antinuclear antibodies (ANA), 15% of anticardiolipin antibodies, 12% of antithyroid antibodies, and 7% of anti-smooth muscle antibodies [123]. The presence of these autoantibodies can be isolated, and these patients generally do not present with signs and symptoms of an autoimmune or connective tissue disease [123].

At present, no definite data are available on the effect of HCV clearance by DAA therapy on all the above non-MC rheumatic disorders.

## 10. HCV and Miscellaneous Associations

Other HCV-associated extrahepatic manifestations include pulmonary fibrosis, licken planus and porphyria cutanea tarda, and thyroid diseases [12,124,125].

### 10.1. HCV and Pulmonary Fibrosis (PF)

The association between HCV infection and pulmonary fibrosis is based on the high frequency of anti-HCV antibodies in individuals with idiopathic PF. Furthermore, HCV patients have a bronchial inflammatory reaction, characterized by lymphocytes and neutrophils. However, the few data in the literature report conflicting results on the association between idiopathic PF and HCV; therefore, there is no clear evidence of a causal association between the two conditions at present. Furthermore, no data are available on the effects of DAA treatment, but HCV clearance may prevent the development of new cases of PF [126].

### 10.2. HCV and Licken Planus (LP)

The association between LP and HCV is based on many epidemiological studies, which have shown a high prevalence (22.3%) of HCV in subjects with LP, albeit with some geographic variability. The studies conducted to verify pathogenetic mechanisms seem to indicate that LP is the result of an immune response to HCV antigens by the host rather than a direct viral action [127]. Different case series on the effects of HCV-clearance by DAA on LP have been published. The data of these studies have shown that, after treatment with DAAs, 82% of cases show a resolution of or improvement in the disease, although some cases may relapse [128,129].

### 10.3. HCV and Porphyria Cutanea Tarda (PTC)

The PCT, in southern European countries, appears to be closely associated with chronic HCV infection. However, PCT can be considered a rare complication of HCV infection. It has been postulated that HCV may have a direct action on the oxidative mechanisms that inhibits uroporphyrinogen decarboxylase in the liver. Antiviral treatment with DAAs has shown that HCV clearance is associated with the PCT healing and the negativization of urinary porphyrins, suggesting a direct involvement of the virus in the active PTC [130].

### 10.4. HCV and Thyroid Disease

The association between thyroid damage and HCV is frequently reported, especially when cryoglobulinemia is present. Autoimmune thyroiditis is frequently associated with HCV infection. From a clinical perspective, autoimmune thyroiditis can be subclinical or present with a picture of hypothyroidism. An association between chronic HCV infection and papillary thyroid carcinoma has also been described. Clearance of HCV by the DAA is the primary approach to prevent the development of thyroid diseases in these patients [131].

## 11. Discussion

There is strong evidence that chronic HCV infection has a significant, direct or indirect impact on the increased incidence of MC, glomerulonephritis, neuropsychiatric manifestations, atherosclerosis and cardiovascular disease, as well as on mortality, due to complications of vasculitis, glomerulonephritis, renal failure and cardiovascular disease. Furthermore, chronic HCV infection causes insulin resistance and a significant increase in type 2 diabetes cases which, in turn, is associated with a higher risk of advanced liver fibrosis, decompensated cirrhosis, HCC and cardiovascular disease. Therefore, in the management of patients with chronic HCV infection, it is necessary to consider a correct strategy for the prevention and control of extrahepatic manifestations that have an important impact on both mortality and health costs.

The recent introduction of new IFN-free DAA treatments that are safe and highly effective in eradicating HCV in about 100% of cases has basically changed the prognosis of these patients. Several studies have shown that clearance of HCV by DAAs reduces both the incidence and severity of HCV-associated extrahepatic manifestations. Elimination of HCV by DAA has been shown to improve CV and reduce the incidence of MC cases; therefore, it is reasonable to expect a decrease in HCV-related cases of MC in the coming years. Patients who have eradicated HCV by DAA therapy but still have MC should be followed up for a long time, as they remain at high risk of developing NHL. Recent data suggest that the eradication of HCV improves atherosclerosis, reduces the incidence of cardiovascular disease and related mortality, improves or reverses insulin resistance, and reduces the incidence of type 2 diabetes. Moreover, HCV clearance significantly improves renal function and the incidence of end-stage renal disease. In addition, DAA treatment appears to improve and prevent some neurological and psychiatric manifestations as well as the quality of life.

Regarding the prevention and regression of HCV-related extrahepatic manifestations, an important aspect to consider is the optimization of the timing of DAA treatment. In this respect, it should be noted that many of the extrahepatic manifestations depend on the duration of HCV infection. Furthermore, the above data show that the advanced stages of extrahepatic disease, e.g., atherosclerotic plaques, advanced kidney disease, diabetes, some established neurological manifestations and NHL, cannot be cured, although some may be stabilized after HCV clearance. Therefore, to optimize therapeutic outcome, DAA treatment should always be started as early as possible in the natural history of HCV infection. An early therapeutic approach not only allows for many of the extrahepatic manifestations that are still in a reversible stage of the disease to be cured, it can also prevent a significant portion of the extrahepatic diseases that develop due to delayed treatment. Therefore, it is necessary for the public health system to implement effective screening strategy to identify unknown cases of HCV infection, in order to treat them at an early disease stage, thus favoring the eradication of HCV, as stated by WHO, reducing the progression and complications of liver diseases, and preventing or treating HCV-related extrahepatic manifestations.

## Figures and Tables

**Table 1:** Most frequent and well-characterized HCV-infection-associated extrahepatic manifestations.

Reported Condition	Affected Systems/Organs
Mixed cryoglobulinemic vasculitis	Immune
Non Hodgkin Lymphoma	Immune
Membrano-Glomerulonephritis type I	Renal
Insulin-resistance	Endocrine
Type 2 Diabetes	Endocrine
Ischemic heart disease with coronary vasculitis	Cardiovascular and circulatory
Coronary artery disease	Cardiovascular
Carotid atherosclerosis	Circulatory
Cognitive impairment	Nervous
Depression	Psychiatric
Pulmonary fibrosis	Lungs
Lichen planus	Skin
Porphyria cutanea tarda	Metabolic disease
Thyroid disease	Thyroid

**Table 2:** DAA therapy in patients (*n*) with hepatitis C virus-related cryoglobulinemic vasculitis.

Authors/ Year	Study Design	PatientsN.	Stage of Liver Status (%)	Clinical Manifestations N. (%)	Antiviral Agent Duration, Weeks (%)	Concomitant Iummunosop-pression N.	SVR after Therapy (%)	Clinical Response	Follow-Up after DAAs (Weeks)
Sise et al., 2016 [16]	Case series	12	CH: 6 (50) C: 6 (50)	Purpura: 6 (50) Arthralgias: 7(58) Neuropathy: 4 (33) Raynaud Phen.: 2 (17) Sicca syndrome: 1 (8) Glomerumlonephritis: 4 (33)	SOF + SIM 12 w: 8 (67) SOF + RBV 12w: 2 (17) SOF + RBV 24: 2 (17)	6 (RTX, PLEX)	10 (83)	CR: 5 (42) PR: 5 (42) NR: 2(17)	12–24
Gragnani et al., 2016 [17]	Prospective cohort	44	CH:27 (61) C: 17 (37)	Purpura: 32 (73) Arthralgia: 26 (59) Weakness: 34 (77) Neuropathy: 28 (63) Raynaud Phen.: 14 (32) Nephropathy: 4 (9) Sicca syndrome: 18 (41) Skin ulcers: 6 (14) NHL: 2 (5)	SOF + RBV 24: 18 (41) SOF + SIM (+RBV): 12 (27) SOF + DAC (+RBV): 4 (32) SOF + LED (+RBV): 10 (23)	9 (RTX) 6 (PLEX) 6 (RTX, PLEX)	44 (100)	CR: 29 (66) PR: 12(42) NR: 3 (7)	6
Saadoon et al., 2017 [18]	Open label prospective cohort	41	CH:23 (46) C: 18 (44)	Purpura: 31 (76) Arthralgias: 26 (63) Neuropathy: 21 (51) Skin ulcers: 7 (17) Nephropathy: 5 (12) Gut involvement: 1 (2) NHL: 1 (2)	SOF + DAC 12 months *n* = 32 (78) SOF+DAC 24 months *n* = 9 (22)	2 (RTX, PLEX)	41 (100)	CR: 37 (90) PR: 4 (10) NR: 0	104
Emery et al., 2017 [19]	Retrospective cohort	18	C: 12 (67)	Purpura:15 (83.3) Neuropathy: 6 (33) Nephropathy: 10 (55) NHL: 6 (17)	IFN/RBV/DAA:7 (39) IFN-free: 11 (61) SOF + RBV: 5 (28) SOF + SIM: 3 (28) SOF + LED ± RBV: 3 (16) 3D + RBV: 2 (11)	4 (RTX, PLEX)	16 (91)	CR: 7 (39) PR: 4 (22) NR: 7 (39)	20
Lauletta et al., 2017 [20]	Prospective cohort	22	CH:16 (73) C:6 (27)	Purpura: 22 (100) Weakness: 22 (100) Arthralgias: 22 (100) Neuropathy: 2 (9) Glomerulonephritis: 4 (18) NHL: 3 (14)	3D: 3 (14) SOF + RBV: 14 (64) SOF + LED: 4 (18) SOF + DCV: 1 (5)	NA	22 (100)	CR 14 (64) PR: 5 (23) NR: 3 (14)	48
Mazzaro et al., 2018 [21]	Retrospective cohort	22	CH:9 (41) C:13 (59)	Purpura: 12 (55) Arthralgias: 12 (55) Neuropathy: 10 (45) Raynaud Ph.: 4 (18) Sicca syndrome: 2 (9) B-cell NHL: 2 (9)	3D: 3 (14) SOF+RBV: 10 (45) SOF+ SIM±RBV: 4 (18) SOF+LED ± RBV: 5 (23)	NA	21 (95)	CR: 14 (64) PR: 3 (14) NR: 5 (23)	48
Passerini et al., 2018 [22]	NA	35	CH:14 (40) C:14 (40)	Purpura: 24 (69) Arthralgia: 6 (17) Neuropathy: 11 (31) Nephropathy: 2 (6) NHL: 1 (3)	SOF-based regimen: 31 (88) 2D regimen: 1 (9) 3D regimen: 3 (8)	NA	35 (100)	CR: 15 (43) PR: 13 (37) NR: 7 (20)	NA-
Bonacci et al., 2018 [23]	Prospective cohort	46	CH:18 (39) C:28 (61)	Purpura: 29 (63) Weakness: 28 (61) Arthralgias: 16 (35) Neuropathy: 19 (41) Nephropathy: 9 (20) Sicca syndrome: 3 (7)	SOF-based regimen: 21 (46) 3D regimen: 13 (28) SIM + DAC: 4 (9) GZB + EBR: 3 (6) PegIFN + DAAs: 4 (9) FDV + LDR: 1 (2)	3 (RTX, PLEX)	46 (100)	CR: 37 (80) PR: 5 (11) No R: 4 (9)	96
Cacoub et al., 2019 [24]	Prospective cohort	148	CH:59 (46) C: 70 (54)	Purpura: 85 (57) Arthralgias: 94 (64) Skin necrosis: 15 (10) Neuropathy: 86 (58) Nephropathy: 25 (17) Hypertension: 44 (30) Severe vasculitis 43 (29) B cell-NHL: 13 (9)	SOF + RBV: 51 (34) SOF + DAC: 53 (36) SOF+ LED: 23 (16) SOF+ SIM: 18 (123)	21 (GC, IS, or PLEX)	141 (97)	CR: 106 (72) PR: 33 (22) No R: 7 (5)	64
Visentini et al., 2019 [25]	NA	45	C 18 (40)	Purpura: 37 (82) Ulcers: 4 (9) Arthralgias: 21 (47) Neuropaty: 34 (76) Sicca syndrome: 15 (33) Nephropathy: 9 (20) NHL: 8 (18)	SOF-based regimen: 45 (100)	NA	45 (100)	CR: 35 (78) PR: 8 (18) NR: 2 (4)	75
Pozzato et al., 2020 [26]	Retrospective cohort	67	CH 25 (38) C 42 (63)	Purpura: 33 (49) Arthralgias: 36 (54) Neuropaty: 28 (42) Sicca syndrome: 3 (4) Raynaud F. 10 (15) NHL: 6 (9)	SOF-based regimen: 52 (78) 3D regimen: 12 (18) Asunaprevir/ daclatasvir: 4 (6)	NA	64 (95)	CR: 40 (60) NR: 27 (40)	48

C, cirrhosis; CH, chronic hepatitis; NHL, non-Hodgkin’s lymphoma; NA, not available; PegIFN, pegylated interferon, RTX, rituximab (protocol, consisting of 4 weekly infusions of 375 mg/m^2^); PLEX, plasmapheresis; SVR, sustained virologic rsponse; CR, complete response; PR, partial response; NR, no response; SOF, sofosbuvir; RBV, ribavirin; SIM, simeprevir; DAC, daclatasvir; LED, ledipasvir; 3D regimen, paritenavir+ ombitasvir+ ritonavir+ dasabuvir; DLR, deleobusvir; GZB, grazoprevir, EBR, elbasvir, FDV, faldaprevir.

**Table 3:** DAA therapy in patients with low-grade non-Hodgkin lymphoma.

Authors, Year	Study Design	N. Pts	Lymphoma Histology	Liver Status N. (%)	Mixed Cryoglobu-linemia	DDA Therapy	Chemotherapy N. (%)	Sustained Virologic Response	NHL Response N. (%)	Follow-Up (Mos.)
Carrier et al., 2015 [40]	Case series	5	MZL: 3 (60) DLBCL: 2 (40)	Chronic hepatitis: 5 (80) Cirrhosis: 1 (20)	MC type II: 3	SOF-based regimen	Concomitant: 3 (60)	5 (100)	CR: 5 (100);	9–12
Alric et al., 2016 [41]	Prospective cohort	10	MZL: 6 (60) DLBCL: 3 (30) Other: 1 (10)	Chronic hepatitis: 9 (90) Cirrhosis: 1 (10)	-	SOF-based regimen	Concomitant: 9 (90)	9 (90)	CR: 9 (90) PR: 1 (10)	12
Arcaini et al., 2016 [42]	Retrospective cohort	46	MZL: 37 (80) LPLm: 2 (4) FL: 2 (4) CLL/SLL: 4 (9) NHL NOS: 1 (2)	Cirrhosis: 7 (15)	-	SOF-based regimen: 39 (85) Other regimen: 7 (11)	No	45: (98)	CR: 12 (26) PR: 19 (41) SD: 11 (24) PD or NR: 4 (9)	8
Persico et al., 2018 [43]	Prospective cohort	20	DLBCL: 20 (100)	Chronic hepatitis: 16 (80) Cirrhosis: 4 (20)	-	SOF-based regimen: 20 (100)	Concomitant: 20 (100)	20 (100)	CR: 19 (95) PD: 1 (5)	12
Occhipinti et al., 2019 [44]	Case series	7	DLBCL: 7 (100)	Cirrhosis: 1 (14)	-	SOF-based regimen: 7 (100)	Before DAA: 2 (29) Concomitant: 5 (71)	7 (100)	CR: 7 (95) PD: 1 (5)	12
Merli et al., 2019 [45]	Retrospective cohort	47	DLBCL: 45 (96) FL: 2 (4)	Cirrhosis: 12 (26) Chronic hepatitis: 35 (74)	-	SOF-based regimen: 47 (100)	Before DAA: 38 (81) Concomitant: 9 (19)	45: (96)	CR: 46 (98) PD: 1 (2)	33.6
Frigeni et al., 2020 [37]	Retrospective cohort	66	MZL:53 (80) Non-MZL: 13 (20)	Chronic hepatitis: 59 (89) Cirrhosis: 7 (11)	-	SOF-based regimen: 66 (100)	No	65 (9)	CR: 14 (21) PR: 31 (47) SD: 15 (23) PD: 5 (8)	17

MZL, marginal-zone lymphoma; DLBCL, diffuse large B-cell lymphoma; LLC, chronic lymphocityc leukemia; FL, follicular lymphoma; LPL, lymphoplasmocytic lymphoma; SOF, sofosbuvir; Other regimen: paritapreveir /ritonavir/ombitasvir+/-dasabuvir+/-ribavirin. CR, complete response; PR, partial response; SD, stable hematological disease; PD, progressive disease; NR, no response.

**Table 4:** Clinical presentations of patients with Cryoglobulinemic nephritis (CN).

Isolated proteinuria (<3 g/24 h), usually with microscopic hematuria (30%).
Nephrotic syndrome (20%)
Acute nephritic syndrome (15%). Some patients present with a mixed nephrotic and nephritic syndrome
Macroscopic hematuria (10%)
Chronic renal insufficiency (10%)
Acute kidney injury with oligoanuria (5%)

## Data Availability

The data that support the findings of this study are available from the corresponding author upon reasonable request.
